# Both high and low levels of cellular Epstein-Barr virus DNA in blood identify failure after hematologic stem cell transplantation in conjunction with acute GVHD and type of conditioning

**DOI:** 10.18632/oncotarget.8803

**Published:** 2016-04-19

**Authors:** Qin Li, Lalit Rane, Thomas Poiret, Jiezhi Zou, Isabelle Magalhaes, Raija Ahmed, Ziming Du, Nalini Vudattu, Qingda Meng, Åsa Gustafsson-Jernberg, Jacek Winiarski, Olle Ringdén, Markus Maeurer, Mats Remberger, Ingemar Ernberg

**Affiliations:** ^1^ Department of Microbiology, Tumor and Cell Biology (MTC), Karolinska Institutet, Stockholm, Sweden; ^2^ Division of Therapeutic Immunology, Labmed, Karolinska University Hospital, Huddinge, Stockholm, Sweden; ^3^ Department of Oncology-Pathology (OnkPat), Karolinska University Hospital, Stockholm, Sweden; ^4^ Public Health Agency, Solna, Sweden; ^5^ Division of Neuropathology, Department of Pathology, Brigham and Women's Hospital, Harvard Medical School, Boston, MA, USA; ^6^ Department of Immunobiology and Internal Medicine, Yale University, New Haven, CT, USA; ^7^ Department of Clinical Science, Intervention an Technology (CLINTECH), Karolinska University Hospital, Huddinge, Stockholm, Sweden; ^8^ Center for Allogeneic Stem Cell Transplantation, Karolinska University Hospital, Huddinge, Stockholm, Sweden; ^9^ Department of Pediatrics, Karolinska University Hospital, Huddinge, Stockholm, Sweden

**Keywords:** stem cell transplantation, EBV DNA-load, T-cell phenotype, total body irradiation, aGVHD, Immunology and Microbiology Section, Immune response, Immunity

## Abstract

The level of Epstein-Barr virus DNA in blood has proven to be a biomarker with some predictive value in allogeneic hematopoietic stem cell transplantation patients (HSCT). We evaluated the impact of EBV load on survival of 51 patients (32M/19F, median age: 32 years, from < 1 to 68 years old), who had received HSCT for different types of malignancies (49 cases) or non-malignancies (2 cases). The overall survival [1]was compared between patients with extreme and moderate cell bound EBV DNA levels. Different sources of stem-cells (peripheral blood stem, *n* = 39; bone marrow, *n* = 9; or umbilical cord blood, *n* = 3) were used. Twenty patients received reduced-intensity conditioning regimen while the other 31 received myeloablative conditioning. Patients with high or very low level of cell bound EBV-DNA levels had a shorter OS than those with moderate EBV load: OS at 5 years was 67% vs 90% (*p* < 0.03). There was a conspicuous relationship between EBV load and the reconstitution dynamics of total and EBV-specific T cells, CD4+ and CD4- CD8- (double negative) T cells in the few patients where it was analyzed. This was not statistically significant. Two other factors were associated to early mortality in addition to high or low EBV load: acute GVHD II-IV (*p* < 0.02) and pre-transplant conditioning with total body irradiation (TBI) ≥6 Gy, (*p* < 0.03). All the patients meeting all three criteria died within two years after transplantation. This points to a subgroup of HSCT patients which deserve special attention with improvement of future, personalized treatment.

## INTRODUCTION

Patients receiving bone marrow (BMT) or hematologic stem cell transplants (HSCT) show a considerable risk to develop EBV-associated post-transplant lymphoproliferative disorder (PTLD) and lymphomas [1]. The following risk-factors have been associated with EBV-related complications after HSCT: HLA and EBV mismatch between recipient and donor, reduced intensity conditioning (RIC), acute graft-*versus*-host disease (GVHD) and pre-transplant splenectomy [2]. PTLD may be prevented or even cured by administration of donor derived EBV-specific cytotoxic T lymphocytes [3, 4]. Thus, imbalance in the control of the persistent, latent EBV-infection is one major factor in the pathogenesis of these complications.

Epstein-Barr virus (EBV) is a gamma herpesvirus with the unique capacity to establish latent infection in human B lymphocytes and also to activate them into proliferating lymphoblasts, acting thereby as a predisposing factor for different types of B-cell malignancies [5, 6]. Primary EBV infection is widely spread and results in lifelong latent infection in more than 90% of immunocompetent adults worldwide [7, 8]. Viral proteins expressed in the latently infected B-cells serve as targets for strong T-cell mediated rejection responses, that can limit the proliferation of EBV carrying lymphoblasts [9]. In addition it has been demonstrated that helper CD4+ T lymphocytes can play an instrumental role in controlling the EBV-latency [10].

Multiple evidence points to an important role of EBV-specific T-cells in the long term control of the EBV carrier state. Failure of this control leads to the occurrence of EBV positive lymphomas in immunosuppressed patients after transplantation and as a late, severe AIDS-determining outcome of HIV infection [11, 12]. We can better understand the critical components of immunity controlling EBV by studying the dynamics of the immune reconstitution during the post-HSCT period. Together with EBV-DNA load as measured in blood features of this reconstitution may provide more precise predictive tools in guiding the post-transplant therapeutic strategy.

EBV DNA levels in blood reflect the intricate and complex balance between EBV and the host, including both EBV replication, the host response to the virus and to virus-infected cells. After transplantation the levels of EBV DNA-load in blood is affected both by immunosuppressive treatment and immune stimulatory mechanisms, like acute graft-*versus* host disease [13, 14]. In BMT-/HSCT-patients the EBV-genomes are predominantly detected in the virus carrying B-lymphocytes, while cell free EBV-DNA in blood/plasma is more rare [14, 15]. In organ transplanted patients and cancer patients also, cell free EBV-DNA can be useful for clinical predictions [16, 17]. After organ-transplantation rapidly rising EBV DNA-levels can reflect or even predict severe complications [18–20]. The predictive value of EBV DNA-load for patient outcome is well-documented in transplanted patients [4, 21]. In BMT and HSCT-patients very high levels of EBV DNA can be detected after certain conditioning, e.g. treatment with anti-thymocyte globulin (ATG) [4], which is also associated with higher risk for PTLD and other adverse complications [16]. The EBV status has been claimed to be of limited value as predictive marker of treatment response in adults with PTLD [22]. The literature is partly confounded by the mix of using non -cell-bound (in plasma) and cell-bound EBV-DNA. For patient follow up or decisions on treatment in individual patients the EBV DNA load has so far been of limited value.

In a clinical follow-up project we determined EBV load and immune parameters in HSCT patients by regular sampling during one year after transplantation. We found a strong prognostic value of predefined levels of EBV DNA load. Patients with very low or high levels of cell bound EBV-DNA in blood early after transplantation showed a poor prognosis, compared to patients with intermediate levels. When combined with two other risk factors, severe acute GVHD (aGVHD II-IV) and conditioning with high dose total body irradiation (TBI) none of these patients survived more than two years after transplantation.

## RESULTS

### Grouping of patients based on EBV DNA load

The EBV genome load in PBMC was followed for 12 months post transplantation. The EBV load values during the first three months were used as the basis for assigning the patients to either of two groups.

Thirty of the 51 patients (60%) were assigned to the EBV_high+low_ group, according to our definition. Twenty-one patients (40%) belong to the EBV_intermediate_ group. There were no difference in clinical parameters between these two groups (Table 1).

**Table 1:** Characteristics of patients and donors

	Whole population	High+Low	Intermediate	*p*-value
***N*** =	51	30	21	
**Age**	32(<1 −68)	32 (<1-68)	34 (7-68)	0.67
**Children** (<18y)	17	9 (30%)	8 (38%)	0.56
**Sex** (M/F)	32/19	21/9	11/10	0.25
**Malignancy/Non-Malignancy**	49/2	28/2	21/0	0.50
**Stage** (early/late)	22/29	12/18	10/11	0.77
**Donor:**				
Sibling/MUD/MM	12/32/7	6/19/5	6/13/2	0.37
**Donor age**	30(0-62)	30 (0-62)	28 (13-57)	0.98
**Conditioning:**				
MAC/RIC	31/20	15/15	16/5	0.08
TBI ≥6 Gy	17	10 (33%)	7 (33%)	1.00
**ATG**	37	21 (70%)	16 (76%)	0.75
**GVHD prophylaxis:**				
CsA+MTX/other	33/18	17/13	16/5	0.23
**Stem-Cell source:**				
BM/PBSC/CB	9/39/3	2/25/3	7/14/0	0.28
**CD34+ cell dose** (x10^6^/kg)		7.1 (0.1-28.2)	5.5 (1.8-22.8)	0.55
**CMV sero neg/neg**	9	4	5	0.46
**EBV sero-MM**	4	3	1	0.63
**aGVHD 0-I/II-IV**	23/28	15/15	8/13	0.26
**Folow-up (months)**	75 (42-103)	57 (42-103)	86 (44-99)	0.25

Early stage; CR1/CP1 or non-malignant disorder, Late stage; beyond CR1/CP1, MUD; matched unrelated donor, MM; mismatched donor, MAC; myeloablative conditioning, RIC; reduced intensity conditioning, CsA; cyclosporine, MTX; methotrexate, BM; bone marrow, PBSC; peripheral-blood stem cells, CB; cord blood, CMV sero neg/neg; cytomegalovirus pre-SCT serological negative donor and recipient, EBV sero-MM; serological mismatch between donor and recipient, aGVHD; acute graft-versus-host disease.

### Overall survival of patients in the two EBV groups

**Figure 1:**
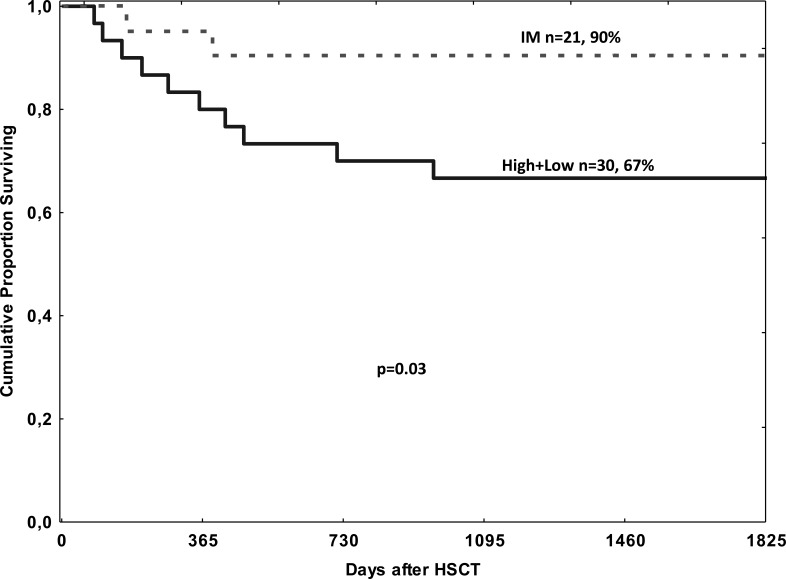
Overall survival depending on EBV DNA levels after HSCT EBV DNA levels were classified into two groups: intermediate (IM) (*n* = 21) or high + low (*n* = 30). The overall survival rates were 90% in EBV intermediate group and 67% in EBV high+low group, respectively; there was a significant difference in the overall survival rate between the two groups (*p* = 0.03).

The EBV_high+low_ patients had a lower overall survival rate than those in the EBV_intermediate_ group (Figure 1; *p* = 0.03). OS at 5 years was 67% *vs* 90%, (*P* < 0.03).

### Risk factors, survival and cause of death

Three factors significantly or close to significantly correlated to mortality in the univariate analysis (*P* < 0.10; Table 2). Another three factors (with *P* < 0.20) were also identified by multivariate analysis. In the final combined multivariate analysis three factors were associated to mortality: high+low EBV DNA load, acute GVHD II-IV and conditioning with TBI ≥6 Gy (Table 2)

**Table 2:** Univariate and multivariate analysis of factors associated to Mortality

Factor	HR	*p*-value	Multivariate
**Intermediate EBV response**	0.22	0.03	0.12, 0.02-0.59, <0.01
**Age**	1.02	0.17	
**Sibling donor**	0.87	0.84	
**CsA+MTX**	1.16	0.80	
**Late stage**	0.58	0.33	
**CD34+ cell-dose**	1.00	0.84	
**Donor age**	1.03	0.17	
**RIC**	0.97	0.96	
**ATG**	1.40	0.61	
**aGVHD II-IV**	3.27	0.07	4.72, 1.25-17.8, 0.02
**PBSC**	1.01	0.98	
**CMV sero neg/neg**	0.93	0.92	
**EBV sero-MM**	2.82	0.18	
**TBI ≥6 Gy**	2.61	0.08	3.55, 1.13-11.1, 0.03

In the multivariate analysis HR, 95% confidence interval and p-value are presented.

Late stage; beyond CR1/CP1, RIC; reduced intencity conditioning, CsA; cyclosporine, MTX; methotrexate, PBSC; peripheral-blood stem cells, CMV sero neg/neg; cytomegalovirus pre-SCT serological negative donor and recipient, EBV sero-MM; serological mismatch between donor and recipient. TBI ≥6 Gy; total-body irradiation ≥6 Gy.

**Figure 2:**
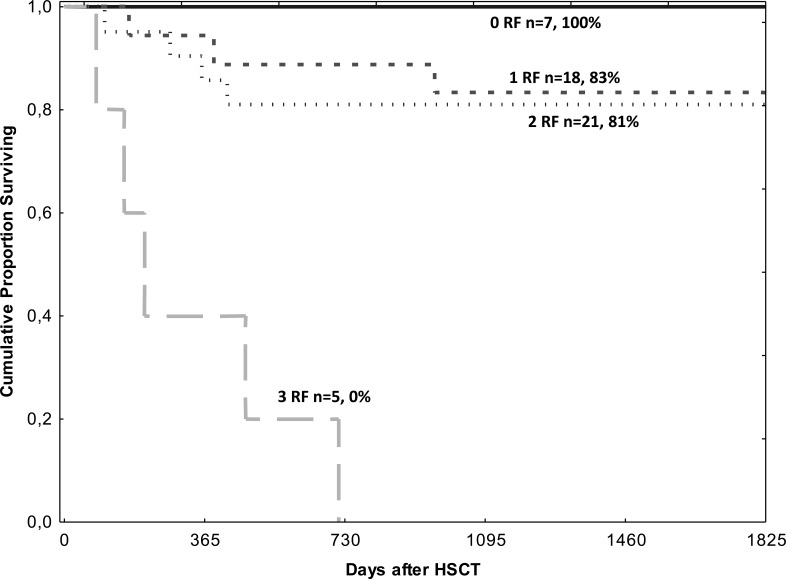
Overall survival after HSCT in patients without (*n* = 7), with one (*n* = 18), two (*n* = 21) and three (*n* = 5) of the risk-factors found in the multivariate analysis The risk-factors were: EBV DNA load low + high, acute GVHD II-IV and conditioning containing TBI ≥6 Gy.

Combined analysis of these risk factors showed that of the 51 patients, those with none of the risk factors (0 RF, *n* = 7) showed a survival rate of 100%; those with one (*n* = 18) had a survival rate of 83%, with two risk factors (*n* = 21) 81% and those with all three risk factors (*n* = 5) showed the worst survival rate, 0% (Figure 2). In addition to the patient group with high or low EBV load (*n* = 30) which by definition had at least one risk factor, 15 patients had two risk factors and five all three risk factors. In the EBV_intermediate_ patient group, seven patients had no risk factor (7/21), eight had one and six had two risk factors (6/21). The causes of death (*n* = 2) in the EBV_intermediate_ patients were bacterial infection (1/21) and cGVHD (1/21). The causes of death (*n* = 11) in the EBV_high+low_ group were specifically relapse (*n* = 4), bacterial infection (*n* = 3), organ failure (*n* = 3, one also with bacterial infection), acute GVHD (*n* = 1) and a secondary malignancy (*n* = 1, not EBV related).

### Dynamics of immune reconstitution in the two EBV load groups

In fourteen of the patients we performed follow up of cellular immune parameters. The analysis included frequency of CD3+ T-cells, CD4+ cells, CD8+cells, Treg cells (CD3+/CD4+/CD4+CD25hi+/Foxp3+/CD127-) and CD4-negative/CD8-negative cells (double negatives, DN). Seven of these patients belonged to the EBV_intermediate_ group and seven to the EBV_high+low_ (5 in EBV_low_ group and 2 in EBV_high_ group).

**Figure 3:**
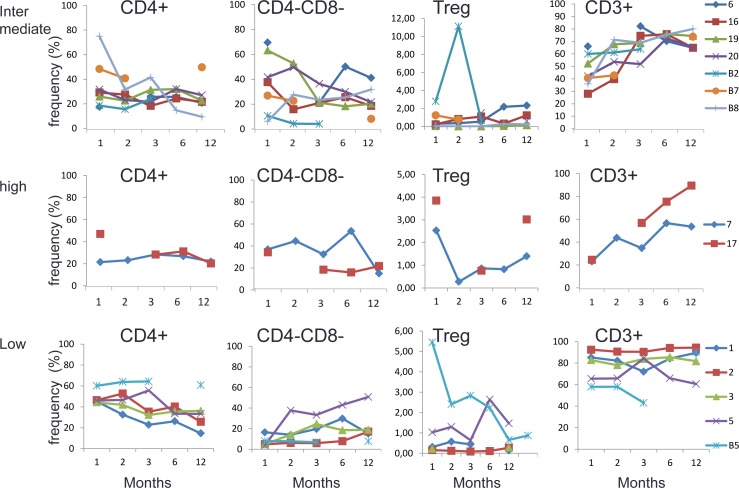
The frequency of CD4+ cell, CD4-CD8- (DN) T cells, Treg cells and CD3+ cells and T_regs_in EBV _intermediate_, EBV_low_ and EBV_high_ groups in 1^st^, 2^nd^, 3^rd^, 6^th^ and 12^th^ month after stem cell transplantation Each curve represents one patient. Lines are not shown between dots with the same color when there are data missing between the dots. B2, B5, B7 and B8 are children and the remaining numbers refer to adult patients. B2, and 2, 7 and 17 represent non-survivors.

During the first three months after transplantation, the patients in the EBV_low_ group showed high levels of T lymphocytes, above or around 60%. In contrast, the two patients with high EBV load showed low CD3+ frequency during the first 3 months, below 60% with the lowest around 20%. The EBV_intermediate_ group had a CD3+ frequency between 30% to 70% (Figure 3).

The frequency of CD4+ T-cells in the EBV_low_ group was also relatively high, mostly above 30%, while those in the EBV_intermediate_ group predominantly showed CD4+ levels below 30% (Figure 3). Although the individual variation of T_reg_ levels was considerable, the groups with high or intermediate EBV loads and lower CD4+ had a higher proportion of Tregs. While the levels of EBV load inversely related to total CD3 and CD4 levels, as for DNs we could detect such a relation only at one month but not later (Figure 3).

**Figure 4:**
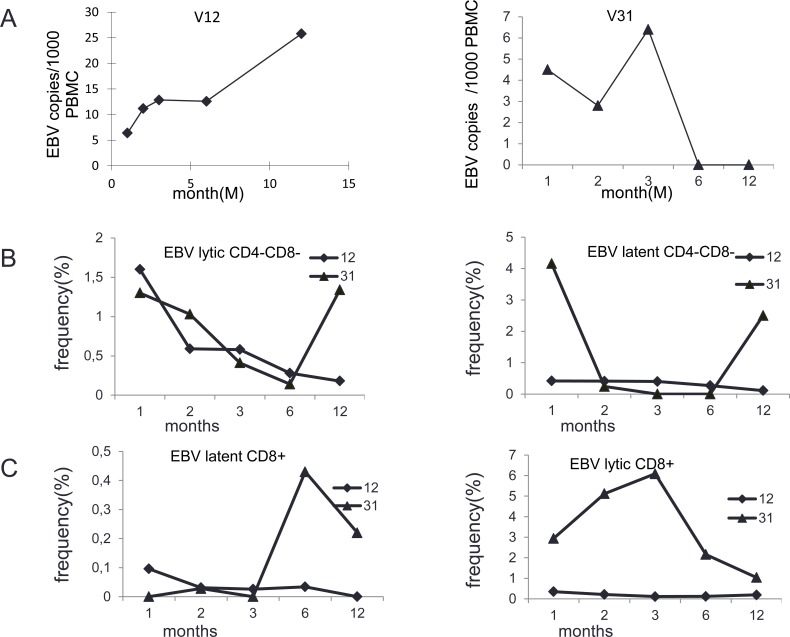
Analysis of EBV specific T cells in patients with different EBV DNA load dynamics **A.** EBV load in adult patients No.12 and No.31 in 1^st^, 2^nd^, 3^rd^, 6^th^ and 12^th^ month after stem cell transplantation. **B.** CD4-CD8- cells specific for lytic EBV antigens (left) and CD4-CD8- cells specific for latent EBV antigens (right) for the same patient at the same time points as in Fig 4A. **C.** CD8+ cells specific for latent EBV antigens (left) and CD8+ cells specific for lytic EBV antigen (right) in the same patients at the same time points after stem cell transplantation as in Figure 4A.

EBV specific CD8+ and DN-cells were measured by labeled HLA- tetramers with specificity for latent and lytic EB viral proteins/antigens. In general, CD8+ and DN cells specific for lytic EBV antigen were more frequent than those with specificity to the latent antigen. Two cases illustrate different patterns of EBV DNA load in relation to reconstitution of EBV specific CD8+ and CD4-CD8- cells (Figure 4). In patient No.12, there was an initial high level of EBV specific T cells which then declined. Opposite the EBV load levels increased after three months peaking at 25780 copies/10^6^ PBMC after the 12 months. In contrast, patient No.31 had high levels of EBV specific CD8+ cells to the lytic viral antigen while the EBV DNA load seemed to be under control.

## DISCUSSION

The crucial role of T cell mediated immune response in controlling the EBV persistent infection is emphasized by the consistent observation that patients with T cell dysfunction are at high risk of developing EBV-associated lymphoproliferative conditions [23, 24]. The EBV DNA levels - EBV load - in blood has been considered a marker which could reflect the functional status of the immune system. In stem cell transplanted patients, the immunosuppressive treatment affects the control of the latent EBV by the immune system. In addition the dramatic immune reconstitution from the donors stem cell transplant poses a stress to the immune system with a time window of immune imbalance early after transplantation.

The EBV DNA load measured the way we do (cell-bound) is likely to reflect indirectly the number of circulating latently infected B-lymphocytes. From other studies we can conclude that there are few if any EBV-infected cells undergoing lytic virus replicayion in the peripheral blood, and we have earlier demonstrated that circulating infected B lymphocytes carry around five EBV-genomes/cell[25].

Viral infections are one common cause of death in SCT patients. Accumulated evidence suggests that high EBV DNA load is one biomarker predicting complications and poor survival. However, this is only true for whole groups, cohorts of high risk patients while correlations have been weak and virtually useless in the individual patient. The surprise from our unbiased analysis is that also low levels of EBV DNA load could be a marker for high risk of mortality. A low level of latent EBV infection during the immune reconstitution early after transplantation may result in an unbalanced control or homeostasis between the virus infection and the host immune system, due to e.g. low trigger/boosting to establish or maintain efficient EBV immune control. Alternatively it might reflect adverse effects of immune suppression or stress of the immune system. The prognostic value of levels of EBV DNA load was enhanced by combination with clinical parameters linked to the conditioning, TBI, or immune suppression, the degree of acute GVHD. The identification of a high risk sub-group of post HSCT patients should put focus on this very specific group and lead to re-evaluation of aspects of conditioning, immune suppression and post-transplant follow up, e.g. the suggested medium-dose etoposide conditioning in SCT due to adult ALL may be more favorable than TBI [26].

The phenomenon of ‘sneaking through’ in tumor biology represents a parallel phenomenon to what we see here. This was described as the ‘the preferential take of tumors after small size inocula to a similar degree with that seen with large size inocula, compared to the rejection of medium sized inocula’[27, 28]. This phenomenon has been reported in several tumor systems [29] as ‘a T cell dependent phenomenon.’ [28]. As high and low EBV DNA load relates directly to the number of EBV carrying cells in peripheral blood, in effect this reflects the exposure of the immune system to high or low amounts of cell associated EBV antigens. This result could be interpreted as a result of high or low dose immune tolerance [30–32]. A correlate to what we observe may be that high EBV DNA levels are strongly associated with the development of post-transplant lymphoproliferative disease [4, 33]. Low EBV load has also in some studies been indicated as a predictor of a poor survival [34].

TBI plays an important role in SCT for both myeloablative regimens and reduced intensity conditioning regimens [35, 36]. It is crucial to find a balanced TBI dose to eradicate the recipient marrow and/or reduce the tumor burden as much as possible and at the same time not to use a high dose that affects the reestablishment of the immune system and a functioning BM. Optimization of the ‘magic dose’ to achieve an ideal balanced treatment would be important. This depends on many factors, e.g., patient age, general condition, malignancy type and degrees, dose/rate/fractionation of TBI, GVHD prophylaxis and stem cell source [36]. In our study, a higher dose of TBI (TBI≥6 Gy) alone was not significantly correlated to OS of the SCT patients, but together with EBV load and the degree of acute GVHD degree, it seems to be a clinical marker to predict poor overall survival.

We made another interesting observation. Two different patterns of dynamics of EBV DNA load were observed, those with a conspicuous peak of EBV load at 1-3 months post transplant which thereafter dropped to stabilize at a low/normal level during 6-12 months - which we designated “type I” dynamics - and those which did not show this distinct early peak of EBV DNA load, but rather developed a variably high and often slowly increasing level of EBV load during 6 to 12 months - “type II”. Originally we hypothesized that there would be a difference in clinical outcome between the patients with these two different patterns of EBV load dynamics. We were unable to establish the significance of such a relation, but so far we have had too few patients with the 2^nd^ type of pattern to possibly reach any significance. One problem with this hypothesis was patients who died early within 6 months after the SCT were then excluded due to that the EBV load could not be followed for longer time up to 12 months. Thus these short term survivors were a priori excluded in our classification of the EBV dynamic patterns. After this failing hypothesis - so far - we decided to use the early levels of EBV DNA load as a basis of grouping patients, which thus also came to include those with the worst outcome according to our analysis.

The limitations of our study are that there are relatively few patients included with different types of donors, different sources of grafts, different types of diagnoses, conditioning, some patients were treated with ATG and others were not, and different types of immunosuppressive prophylaxis was given. Therefore, the findings have to be taken with caution. However, despite these shortcomings, EBV adds a significant impact on survival and the patients with three defined risk-factors had an extremely poor outcome. EBV load may be a surrogate marker for defining patients with poor immune reconstitution after HSCT. Such patients may be at risk to acquire other infections. For instance, these patients may more often have concomitant reactivation of cytomegalovirus (CMV), which has additional immunosuppressive effect [37]. Patients with CMV infections also have an increased risk of bacterial and invasive fungal infection after HSCT [38]. Earlier we have suggested EBV as a good surrogate marker for immune reconstitution after cART treatment in HIV patients [39, 40]. Our data suggest that cell-bound EBV DNA load is an interesting reflection of the quality and balance of reestablishing the immune system. Moderate levels of EBV DNA load, reflects a balanced reconstitution, the parameters of which now should be better established. This will require larger sample size and extensive immune-phenotyping.

## MATERIALS AND METHODS

### Patients

The characteristics of the patients are summarized in Table 1. Blood samples were collected from the patients at 1, 2, 3, 6 and 12 months after HSCT. Fifty-one patients were included, with blood samples from all the three early time points, one, two and three months. Seventeen were children (≤ 18 years old) and 34 were adults. Forty-nine of these underwent HSCT due to malignant disorders. Thirty-two patients received grafts from matched unrelated donors (MUD), seven from HLA-mismatched unrelated donors (MM) and 12 from HLA-identical siblings. Four had a mismatch of EBV status between donor and recipient, one with an EBV negative donor and the other three were EBV negative recipients, as determined by EBV serology. The sources of stem cells were either peripheral blood stem cells (PBSC, *n* = 39), bone marrow (BM, *n* = 9) or umbilical cord blood (CB, *n* = 3).

### Ethics statement

The study was approved by the Stockholm Ethical Committee South 2010/760-31/1. In the case of children consent was also obtained from parents or legal guardians (on file at Center for Allogeneic Stem Cell transplantation, CAST, Karolinska University Hospital, Huddinge).

### Conditioning for the HSCT regimen

The details of conditioning and post-transplant supportive care have been reported elsewhere [1, 41, 42].

Conventional myeloablative conditioning was given to 31 patients and consisted of cyclophosphamide (Cy) at 60 mg/kg for two days in combination with fractionated TBI (FTBI) at 3 Gy/day for four days (*n* = 8), or busulphan (Bu) at 4 mg/kg/day for four days (*n* = 16), or Bu and melphalan 140 mg/m^2^ (*n* = 3)(20). Three patients received FTBI and vepecide 60 mg/kg and one patient received fludarabin (Flu) at 30 mg/m^2^ for 4 days in combination with Bu at 4 mg/kg/day for two days and thithepa 20 mg/kg. Reduced-intensity conditioning (RIC) was given to 20 patients and consisted of Flu at 30 mg/m^2^ for 3-6 days in combination with either Bu at 4 mg/kg/day for two days (*n* = 5), FTBI at 3 Gy/day for two days and Cy at 60 mg/kg/day for two days (*n* = 6), Cy at 30 mg/kg/day for two days (*n* = 1), treosulphan at 12-14 g/m^2^/day for 3 days (*n* = 7), or TBI (2 Gy) (*n* = 1).

### Purification of peripheral blood mononuclear cells

The purification of PBMCs has been described elsewhere [1, 41]. Briefly, five to 10 ml of blood were collected from all patients starting from the fourth week after BMT and then at 2, 3, 6 and 12 months after transplantation. PBMCs were isolated by separating heparinized blood on a Ficoll Hypaque gradient (Amersham Pharmacia, Uppsala, Sweden). The cells were preserved in fetal bovine serum (FBS) containing 10% DMSO a −160°C. Aliquots of PBMCs were thawed and used for analysis of lymphocyte subsets by 12-color flow cytometry and for preparation of DNA for determination of EBV-DNA genome and analysis of viral gene expression.

### DNA extraction and EBV load measurement

DNA was extracted and purified by QIAamp DNA Mini Kit (QIAGEN GmbH, Germany) according to the manufacturer's instruction. The concentration of the purified DNA was measured using NanoDrop ND-1000 spectrophotometer.

The qPCR assay was performed according to Kimura et al [43]. Briefly DNA from the EBV positive Burkitt's lymphoma Namalwa cell line was used to establish a standard curve. The primers for PCR were from the BALF5 EBV gene encoding the viral DNA polymerase [44] The EBV unique upstream and downstream primer sequences were 5′;-CGGAAGCCCTCTGGACTTC-3′; and 5′;-CCCTGTTTATCCGATGGAATG-3′;, respectively (Life Technologies Europe, Stockholm, Sweden). Fluorogenic probe for BALF5 (5′;-TGTACACGCACGAGAAATGCGCC-3′;) and for Albumin (5′; CCTGTCATGCCCACACAAATCTCTCC -3′;) with a sequence located between the PCR primers respectively were synthesized by PE Applied Biosystems (Foster City, USA.). Taqman genotyping master mix was applied (Applied Biosystems, Foster City, USA). The PCR reaction was performed in MicroAmp optical 96-well reaction plate (Applied Biosystems, Singapore) with MicroAmp optical adhesive film (Applied Biosystem, Foster City, USA). Real-time fluorescence measurements were performed on a 7500 Sequence Detector (PE Applied Biosystems). Albumin was used as an internal control.

### Flow cytometry

The analysis of PBMCs was also described previously [41]. Frozen PBMCs from were thawed and 1 × 10^6^ cells were incubated at 4°C for 15 min with the following antibodies: PerCP conjugated anti-CD3 (SK7), APC-Cy7- conjugated anti-CD8α chain (SK1) purchased from BD Biosciences (Stockholm, Sweden), Krome Orange-conjugated anti-CD4 (13B8.2), FiTC-conjugated anti-CD8β chain (2ST8.5H7), as well as antibodies to CD25hi, Foxp3 and CD127 were purchased from Beckman Coulter (Marseille, France). After washing with 1 mL of PBS containing 0.1% BSA, the cell pellet was resuspended in 200 μl of PBS (with 0.1% BSA) and the cells were analyzed by flow cytometric with Navios flow cytometer (Beckman Coulter, Miami, FL, USA) and data analysis was done with software FlowJo (Tree Star Inc., Ashland, OR; USA).

### Grouping according to the EBV load

After determination of EBV DNA levels the patients were divided into two groups: those with intermediate levels of EBV DNA load and those with high or low. The border values between the groups were chosen arbitrarily based on the data, as follows: high EBV load was defined as higher than 90 000 copies/10^6^ PBMC in at least one of the three samples, or higher than 60 000 copies/10^6^ PBMC in at least two of the three samples of the PBMCs collected at one, two and three months after the transplantation. Low EBV load was defined as a genome copy number below 6000 copies/10^6^ PBMC in all the three samples, or negative in at least 2 of the 3 samples. These high or low EBV load groups have then been treated as one group: the EBV_high+low_ group. The remaining cases were defined to have intermediate EBV load (EBV_intermediate_).

### Tetramers for analysis of EBV-specific T cells

Tetramer-guided analysis of EBV-specific T cells was carried out as described earlier [1, 45]. In brief, frozen PBMCs were thawed, washed and incubated with tetramers at 37°C for 30min. The PE labelled HLA-A*0201 tetramers for EBV BMLF-1 (GLCTLVAML) and LMP-2 (CLGGLLTMV) and HLA-A*24 tetramers for BRFL-1 (DYCNVLNKEF) and EBNA-3 (RYSIFFDY) were applied (Beckman Coulter). The cells were washed with staining buffer (PBS with 2% FCS) and incubated at 4°C for 15 min with monoclonal antibodies for cell surface markers, PerCP-conjugated anti-TCR[alpha][beta] (WT31; BD Biosciences), APC-Alexa-Fluor 750-conjugated anti-CD8[alpha] chain (T8; Beckman Coulter) and Pacific Blue conjugated anti-CD4 (Beckman Coulter). The cells were then washed, re-suspended in staining buffer and data acquisition was performed by FACSAria Flow cytometer (BD Biosciences). Finally the data was analyzed with the software FlowJo (Tree Star Inc., Ashland, OR).

### Statistics

Overall survival was calculated using the Kaplan-Meier method and compared with the log-rank test. Uni- and multivariate analysis of factors associated to survival was performed with the Cox proportional hazards model. Factors with a *P*-value < 0.2 in the univariate analysis were included in the backwards elimination multivariate analysis. Factors analyzed are displayed in Table 2. Continuous variables were compared with the Mann-Whitney test and categorical variables with the Fisher exact test. Analysis was performed with the Statistica software (Statsoft, Tulsa, MN, USA).
